# Development of a Cardiac Point-of-Care Ultrasound Curriculum for Anesthesia Residents in Brazil: It is Time to Act.

**DOI:** 10.24908/pocus.v6i2.14780

**Published:** 2021-11-23

**Authors:** Fabio De Vasconcelos Papa, Luiz Guilherme Villares Da Costa

**Affiliations:** 1 Dalla Lana School of Public Health, University of Toronto; 2 Takaoka Anesthesia, Albert Einstein Hospital Sao Paulo Brazil

**Keywords:** point-of-care ultrasound, cardiac ultrasound, hemodynamic instability, ultrasound curriculum

## Abstract

Although the use of cardiac point-of-care ultrasound in anesthesia is well established, with strong evidence supporting its benefit while managing hemodynamically unstable patients during the perioperative period, there is a lack of standardized curriculums incorporating this diagnostic modality as part of the anesthesia residency training. This report aims to describe a FOCUS curriculum based on adult learning theories, and to suggest its implementation as part of the anesthesia residency training considering the learners’ (i) previous experience with ultrasound, (ii) level of training in anesthesia, (iii) and other challenges that can impact the organization and delivery of this project.

## Description of curricular problem

The use of ultrasound in anesthetic practice is already well established such as in regional nerve blocks 1, central venous access 2, and perioperative transesophageal echocardiography 3. 

Recently there has been a great increase in interest and the dissemination of the point-of-care ultrasound technique (POCUS) in the areas of intensive care, surgery, and emergency medicine, confirming that its use in perioperative medicine has a much broader potential, both to improve hemodynamic monitoring and for early diagnosis and management of complications 4. 

Its use in the perioperative period is specifically well established in the following segments: (1) cardiac, (2) pulmonary, (3) hemodynamic assessment, (4) abdominal, (5) vascular access, (6) airway, and (7) assessment of intracranial pressure. 

As a POCUS modality, focused cardiac US (FOCUS) is defined as the use of US at the bedside to evaluate the unstable patient and, within a specific list of diagnoses, individualizing the clinical treatment for a given situation based on the findings using binary and qualitative questions (yes/no). Its use by the anesthesiologist in the perioperative period is related to lower rates of complications and mortality in high-risk patients 5.

The advent of POCUS use in the anesthetic practice has introduced a new set of knowledge and skills, including knowledge of sonoanatomy and the skills of ultrasound scanning, image acquisition, interpretation, and transformation of a three-dimensional structure into a two- dimensional image. 

In terms of training, there is no current consensus on what perioperative FOCUS comprises and no national curriculum in Brazil. The only published national ultrasound curriculum is from the Association of Anaesthetists of Great Britain and Ireland and the Intensive Care Society 6 being directed, without distinctions, to both anesthesia and intensive care trainees.

In order to acquire new skills to perform the FOCUS technique, there is a need to develop a training curriculum that considers the different training phases (handling of the device and acquisition of images, interpretation of images, and clinical correlation). In order to move from one phase to the next, the student needs to demonstrate proficiency in the previous phase. For this reason, there is still much debate around the creation of a training model for anesthesia residents so they can become more proficient and comfortable in performing the examination. It should also be considered the different phases of training, development, and experience within the anesthesia residency program and what is the best time (if any) in the residency program for the introduction of this curriculum.

The first specialty to introduce FOCUS in clinical practice was emergency, followed by intensive care medicine. In 2015, the International Federation of Emergency Medicine (IFEM) released a consensus document to guide the development of FOCUS training programs in Emergency Medicine 7. According to this guide, despite having a more generic character, creating a training program should be based on the development of a basic program and a more advanced one regarding ultrasound application. After that, the training is based on four distinct phases: introduction to the application, development of experience, achievement, and maintenance of competence. 

Recommendations regarding intensive care training are based on the American College of Chest Physician Statement for Critical Care Ultrasonography. Like what has been described in emergency medicine, training is also composed of four different steps: introductory training, portfolio completion, competency assessment, and maintenance of competence 8. 

Regarding training and education in FOCUS for anesthesiologists, despite many initiatives to create a formal curriculum, none has so far managed to be adopted as the gold standard. They are all very similar in the way they are built and in the steps that students must follow to advance in training 9.

In order to learn the FOCUS technique, there must be an integration of two essential factors: the learning of technical skills (acquisition, image optimization) and the interpretation and integration of the findings with the clinical picture. Given these two learning objectives, developing a curriculum based on Constructivist Social Theory associated with a teaching approach for the acquisition of procedural skills has the ideal components for learning the technique.

## Adult Learning Theory

There are many different adult learning theories, but they are all derived from the same concept that the way adults learn is different from how children learn. 

The term Adult Learning Theory or Andragogy introduced by Malcolm Knowles 10 describes how the adult learning process differs from that of children, and it is based on five assumptions: 

1. Adults are independent in their learning process. 

2. They bring broad experience to the new learning process. 

3. They value learning with practical results. 

4. They are more interested in problem-centered approaches. 

5. They bring an internal motivation to learn something new. 

Constructivism is one of those learning theories based on the idea that learners actively construct or make their knowledge using their previous experience as a foundation and build on it with new things that they learn. There are many specific elements and principles of constructivism that shape the way the theory works and applies to students 11: 

6. Knowledge is personal. 

7. Motivation is key to learning. 

8. Learning is a social activity. 

9. Learning is an active process. 

10. Knowledge is constructed. 

11. Learning is contextual. 

 In constructivism, the teacher act as a facilitator and not as a transmitter of knowledge. The primary idea of this theory is that learners construct their knowledge based on what they already know, posing emphasis on the active learning process. 

The application of Constructivism to develop a new model of FOCUS training is based on some of the principles that make it the ideal theory for the development of a training model for anesthesia residents:

- Learner's "construct" their knowledge based on what they already know:

Anesthesia residents (especially during the final years of their training) can integrate different information regarding acute and unstable clinical problems faced during the perioperative period and have enough knowledge to adopt the concept of FOCUS into their clinical practice.

- The teacher is viewed not as a transmitter of knowledge but as a guide who facilitates learning.

Constructivism focuses on the collaborative nature of learning. Knowledge develops from how people interact with each other, their culture, and society at large. Students rely on others to help create their building blocks and learning from others helps them construct their knowledge and reality. 

Vygotsky created two concepts that help to understand how constructivist social theory is used and what is the role of the teacher in this process: Zone of Proximal Development (ZPD) and Scaffolding 12.

- Zone of Proximal Development (ZPD): Vygotsky consistently defines the zone of proximal development as the difference between the current level of cognitive development and the potential level of cognitive development. He maintains that students can reach their learning goals by completing problem-solving tasks with their teacher or engaging with more competent peers. Vygotsky believed that a student would not reach the same level of learning by working alone. As a student leaves his zone of current development, he travels through the zone of proximal development towards his learning goal.
- Scaffolding: To help learners achieve independence, Vygotsky outlined scaffolding as a tool for growth. Learners complete small, manageable steps to reach the goal. Working in collaboration with a skilled instructor or more knowledgeable peers helps students make connections between concepts. As learners grow within their zone of proximal development and become more confident, they practice new tasks with the social support that surrounds them. Vygotsky maintains that learning occurs through purposeful, meaningful interactions with others.

Acquisition of procedure skills is an essential element in health professions education 13. Traditionally, procedures were taught using a "see one - do one" approach, meaning that a teacher demonstrates and describes a procedure, and afterward, the students are asked to practice the same procedure. Although this approach has been prevalent and is still used in some situations, it is an unsystematic and unstructured approach not following the current principles of adult learning and sometimes putting the patient at risk. A more recent approach described by Walker and Peyton describes a stepwise teaching approach consisting of four different steps 14: demonstration, deconstruction, comprehension, and performance. As demonstrated in a systematic review and meta-analysis by Giacomino et al. 15, it proved to be more effective in teaching and acquiring new procedural skills.

## Curricular solution

The creation of a training model based on the Constructivism Theory considers the basic knowledge of the anesthesiologist in training regarding the use of ultrasound for central venous access and regional nerve blocks and extrapolates it for use with the goal of point-of-care for diagnosis and management of common causes of hemodynamic changes in the perioperative period. It also should take into consideration the importance of the concept of ZPD and scaffolding with the instructor having the role of creating different stages of learning and training in order to gradually deepen the technical and theoretical knowledge of the student, avoiding the cognitive load but at the same time to help them to acquire independence in the realization of the technique and interpretation of the results. 

In terms of technical skills acquisition, the student must observe the performance of the correct examination technique performed by the proficient anesthesiologist in an environment free from the pressure generated by an unstable patient, where the result of this assessment will change the clinical approach. An initial phase of varying duration where training is carried out in a simulation environment can offer the ideal opportunity to develop technical and cognitive skills related to the technique. In addition, it is essential to consider in the development of the training program the concept of cognitive load to break the process into small parts (weekly) for a better learning experience. 

Based on all the concepts exposed in the sessions above, the curriculum for training anesthesia residents in the FOCUS modality should be based on a horizontal program to be applied to all anesthesia residents, respecting the different training stages and composed of: 

Phase 1: Program of formal classes with topics related to image acquisition and optimization, handling the device, choice of the probe, and most common clinical scenarios found in the operating room (myocardial infarct, tamponade, pulmonary embolism, hypovolemia). After the student participates in the entire class program and is theoretically assessed (MCQ) with a satisfactory result, he/she would be able to progress to the second training stage. 

Phase 2: Acquisition and interpretation of images performed on the simulator to familiarize the student with the handling of the device and what to expect from the technique. After training in the simulator and proficiency assessment, they could progress to phase 3. 

Phase 3: Final part of the training consisting of examinations performed on actual, low risk and stable patients undergoing elective surgical procedures under the direct supervision of the instructor. If proficiency in the technique is demonstrated and after a logbook of 30 exams (as suggested in the literature 16), there will be a progression towards the realization of the exam in unstable patients with the correlation of the exam findings and decision making.

Consideration needs to be made about placing residents of different years at the same level of training. Although there is a significant difference of experience between the resident in the first and last year, teaching this diagnostic modality is valid from the beginning of the residency and improves patient care. Over time and with new classes of residents starting, the more experienced ones (having had training from the beginning) will be able to facilitate members to learn more junior residents.

Some challenges can threaten the successful implementation of the proposed curriculum: 

1. The limited number of anesthesiologists proficient in the FOCUS technique willing to participate in the training program. 

2. Lack of formal knowledge in simulation, learning theories, and feedback by the anesthesiologists involved. 

3. Need for protected time (outside the operating room) for teaching the technique by both anesthesiologists and residents. 

4. Absence of a worldwide formal training program in the FOCUS technique for the anesthesia specialty. 

5. Although the literature suggests between twenty-five and thirty exams performed under direct supervision, there is no consensus regarding the minimum number of exams after which the student can be considered proficient and able to perform the exam and make clinical decisions based on the findings 16.

## Conclusion

The development of a training program aimed at the anesthesia resident must consider the particularities of the specialty (routine within the operating room) and the type of situations that can be encountered in daily clinical practice (e.g., hemodynamic instability that requires diagnosis and immediate treatment). Despite the importance of the topic, there is no current consensus on what FOCUS training comprises and no national curriculum in Brazil (or elsewhere). 

The adoption of the constructivist model for the development of a training program is based on the fact that the anesthesia resident already has some knowledge regarding the use of ultrasonography (either for performing peripheral blocks or obtaining venous access) and can use it as a starting point to learn this new technique. In addition, the instructor can act as a facilitator of this learning using the concepts of ZPD (Zone of Proximal Development) and Scaffolding described by Vygotsky, creating different stages that must be finalized in order to progress to a more advanced stage, culminating in independence for the performance, interpretation, and integration of the examination findings. 

The goal of this paper was to describe a training model based on the Constructivist Learning Theory (based on the anesthesia residents' previous knowledge of ultrasound) to facilitate, optimize and organize the learning of the focused cardiac ultrasound point-of-care technique based in a program composed of theoretical and practical components in order to familiarize the student with the concept and importance of the technique, progressing towards the development of new skills for the exam, culminating in the interpretation of the results and integration with the clinical picture found aiming at the decision making with the objective of better care to the hemodynamically unstable patient.

## Disclosures

None
